# Wavelength- and Angle-Selective Photodetectors Enabled by Graphene Hot Electrons with Tamm Plasmon Polaritons

**DOI:** 10.3390/nano13040693

**Published:** 2023-02-10

**Authors:** Cheng-Han Huang, Chia-Hung Wu, Rashid G. Bikbaev, Ming-Jyun Ye, Chi-Wen Chen, Tung-Jung Wang, Ivan V. Timofeev, Wei Lee, Kuo-Ping Chen

**Affiliations:** 1 Institute of Photonic System, National Yang Ming Chiao Tung University, 301 Sec. 2, Gaofa 3rd Road, Tainan 711010, Taiwan; 2 College of Photonics, National Yang Ming Chiao Tung University, 301 Sec. 2, Gaofa 3rd Road, Tainan 711010, Taiwan; 3 Kirensky Institute of Physics, Federal Research Center KSC SB RAS, 660036 Krasnoyarsk, Russia; 4 Siberian Federal University, 660041 Krasnoyarsk, Russia; 5 Institute of Imaging and Biomedical Photonics, National Yang Ming Chiao Tung University, 301 Sec. 2, Gaofa 3rd Road, Tainan 711010, Taiwan; 6 Institute of Photonics Technologies, National Tsing Hua University, Hsinchu 30013, Taiwan

**Keywords:** 2D material, Tamm plasmon polariton, distributed Bragg reflector, graphene, photodetectors, wavelength and angle selectivity, LiDAR

## Abstract

Recently, two-dimensional materials have attracted attention owing to their special optical characteristics and miniaturization, with low thickness as well as extremely high responsivity. Additionally, Tamm plasmon polariton (TPP) resonance can be observed by combining a metal film and a one-dimensional (1D) photonic crystal (PC), where an electric field confinement is located at the metal–1D PC interface. In this study, a graphene layer combined with a TPP is proposed as a wavelength- and angle-selective photodetector. The graphene layer is located where the strong field confinement occurs, and the photocurrent response is significantly enhanced with increasing absorption by over four times (from 62.5 μA⋅W^−1^ to 271 μA⋅W^−1^ and undetected state to 330 μA⋅W^−1^ in two different samples). Moreover, the graphene–TPP photodetector has wavelength and angle selectivity, which can be applied in LiDAR detecting, sun sensors, laser beacon tracking, and navigational instruments in the future.

## 1. Introduction

Light detection and ranging (LiDAR) is a widely used technology that emerged after the invention of the laser in the 1960s [1]. Ground-based LiDAR is viewed as a significant component in various applications, such as automotive vehicles [2], artificial-intelligence-based robots, unmanned aerial vehicles [3,4], industry-oriented applications of self-driving cars [5], and robot vision in manufacturing factories. In addition, iPhones and iPads from Apple, Inc. and dynamic indoor motion sensors from Microsoft Corporation [6] employ LiDAR sensors for virtual reality displays. However, there is a lack of effective solutions for LiDAR imaging systems used in automobiles in terms of performance efficiency, component availability, detector size, and cost. Recent advancements in nanophotonics have been considered for replacing conventional LiDAR systems, particularly in applications that require a miniaturized footprint, such as in chip-scaled optical arrays [7,8] and plasmonic optical devices based on metasurfaces [9,10]. Furthermore, a LiDAR structure based on nanophotonics platforms can offer optimized detector efficiencies in the light of strong field confinement in the structure and the characteristics of metasurfaces [11].

Two-dimensional (2D) material with atomic-scale thicknesses, such as transition-metal dichalcogenides (TMDCs), black phosphorus, and graphene, have been introduced in a variety of applications [12,13,14], including nanophotonic and optoelectronic devices such as photodetectors [15], biosensors [16], and absorbers [17]. In addition, photodetectors combined with semiconductor materials such as silicon [18,19], germanium [20,21], and III–V semiconductors [22] have also shown great potential for attractive optical and electrical performance. Although silicon-based photodetectors offer the advantages of high yield and low cost, they are bulky and require complex epitaxial and fabrication processes, which hinders their flexibility when used in nano-plasmonic structures [23,24]. Additionally, a narrow working range can be used for practical applications because of the bandgap of the semiconductors or TMDCs. By contrast, graphene-based photodetectors have received considerable interest [25,26] by virtue of their many advantages, such as a gapless band structure, light absorption in the broad range of 300–2500 nm, and transformation of photons into carriers. The high carrier mobility of graphene enables a very fast response in photovoltaic (PV) conversion, where photons are converted into electric signals. Owing to the atomic layer thickness of graphene, the unique mechanical and optical flexibility of plasmonic nanostructures, with a strong localized field, makes them suitable for optoelectronic devices.

The PV [27], photothermoelectric (PTE) [28], bolometric (BOL) [29], photogating effects [30], and the plasma-wave-assisted mechanism [31] are different mechanisms observed in graphene photodetectors. In PV, the separated photon-induced electron–hole pairs generate a photocurrent. The separation can be caused by either a built-in electric field or an external bias voltage [32,33]. The PV photocurrent is controlled by the junction in the graphene, and the direction of the photocurrent depends on the direction of the electric field. Graphene can be modified as n-type or p-type either by applying a gate voltage in the graphene field-effect transistor (FET) [34] or by placing metals with different work functions underneath the graphene [35]. The influence of the carrier recombination is so high that the photocurrent induced by the internal field cannot be observed for long channel lengths (>0.2 μm) [36]. A higher electric field created by an externally applied bias promotes the collection of carriers at the terminal electrodes, thereby producing a direct current (DC) current. Nonetheless, the PV conversion efficiency of graphene is not good enough because a monolayer graphene can only absorb approximately 2.3% of the incident light. Luckily, efficiency can be optimized if strong localized fields are properly designed. Some studies have demonstrated several solutions, including integrating graphene with an optical cavity [37], a photonic crystal (PC) cavity [38], and a silicon waveguide [39]. The PV conversion efficiency of graphene can be enhanced by transferring graphene onto plasmonic nanostructures, and field confinement can be attained using localized surface plasmon polaritons (SPPs) [40,41].

Plasmonic resonance is a well-known plasmon state that includes SPPs. It creates strong localization of electric fields and has been extensively studied for various applications, including nanoantennas [42], metasurfaces [43], and biosensors [44]. Another type of plasmon state, Tamm plasmon polaritons (TPPs), has been explored recently. A Tamm plasmonic (TP) structure, which was first investigated by Shelykh et al. in 2007 [45], is constructed from a distributed Bragg reflector (DBR) and a metal film. Special optical characteristics can be detected in TPPs, including sharp resonance (high *Q*-factor) and strong field confinement located at the DBR-metal interface. Compared with SPPs, TPPs can be directly excited without any coupler or prism; likewise, the structure of TPPs is more easily fabricated by an evaporation system than that of SPPs because of its layer-by-layer structure. Moreover, TPP resonance can be adjusted by changing either the thickness of the dielectric layers of the DBR or the incident angle of the light source, which is a promising utilization for narrow band filters [46], thermal emitters [47,48], and lasers [49,50].

In this study, an idea was developed for a graphene photodetecting device employing the TP structure. As graphene was transferred onto the DBR, a metal film was fabricated on the graphene layer. The proposed graphene–TPP photodetector was devised and fashioned using gold electrodes to collect electrical signals. The graphene layer does not affect the TPP resonance thanks to the atomic layer thickness of graphene; however, the graphene locates the localized electric field formed by the TP structure. The structure converts incident light into TPPs that are collected and transferred into photocurrents by the graphene layer. The photocurrent generation mechanism of our device is related to the PV effect. In our proposed graphene–TPP photodetector, the photoresponse of graphene can be enhanced by the localized electric field, and the device facilitates angle and wavelength selectivity by means of the TP structure. Our device can be utilized as a miniaturized LiDAR detector for suitable applications, lowering the processing cost, and optimizing the processing yield.

## 2. Materials and Methods

### Graphene Wet Transfer

Single-layer graphene grown on a Cu foil using the chemical vapor deposition technique was transferred using the wet chemical method. Initially, the graphene was covered with a thin layer of photoresist (PMMA-A4) by spin coating. The graphene/photoresist stack was removed after the Cu foil was etched away in a Fe(NO_3_)_3_ solution (33 wt.%) at room temperature (23–26 °C) for approximately 12 h. Subsequently, the stack was cleaned for 1 h with deionized (DI) water to wash off the residual etchant. After the floating stack became etchant-free, it was transferred to the target substrate, which was treated with UV-Ozone prior to the transfer for achieving better quality graphene. The UV-Ozone treatment enhanced the surface energy, which decreased the contact angle, resulting in a free adjustment of the stack on the substrate. Following the transfer process, the sample was left to dry in an oven at room temperature until complete dehydration was achieved. The sample was immersed in acetone to remove the photoresist and subsequently rinsed with isopropanol (IPA).

## 3. Results

Two graphene–TPP photodetecting devices were designed to prove that our device could work in both the near-infrared (NIR) and visible light regions. In Section 3.1, an angle-selective device that works in the NIR region (Device A) is introduced, and a wavelength-selective device that works in the visible light region (Device B) is introduced in Section 3.2.

### 3.1. Angle Selectivity (Device A)

**Figure 1:**
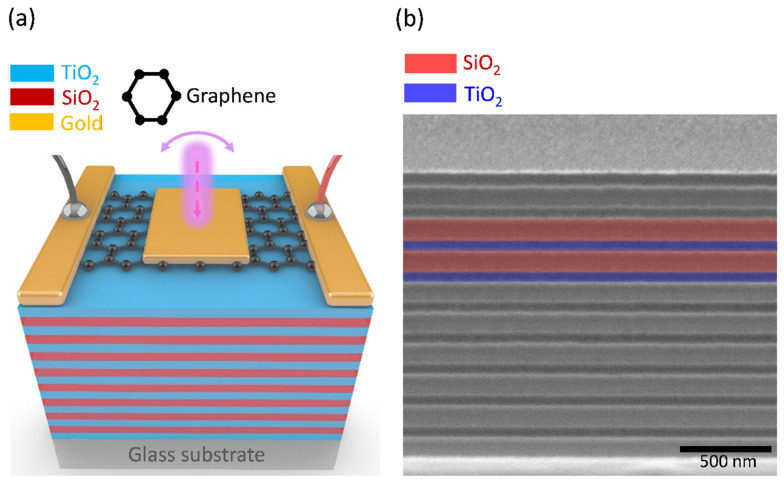
(**a**) A schematic of the graphene Tamm plasmon polariton photodetector (Device A, NIR region sample). (**b**) Scanning electron micrograph of the dielectric layers of the DBR (Device A, NIR region sample).

Figure 1a shows the schematic of the proposed graphene–TPP photodetector. The device was constructed by stacking a glass substrate, alternating dielectric layers of the DBR, transferred graphene, and deposited TPP metal film (35 nm gold) and gold electrodes on top of each other using an E-beam evaporation machine. The graphene channel is where the strong electric field confinement caused by the TP structure is located. The SEM image of the cross-section of the dielectric layers in our DBR is shown in Figure 1b; the thickness of all the silicon dioxide (SiO_2_, refractive index *n* = 1.475) layers is approximately 144 nm, and that of all the titanium dioxide (TiO_2_, refractive index *n* = 2.237) layers is about 95 nm, giving rise to the central wavelength of the DBR at 850 nm, which could be considered as an NIR-region sample. (For the schematic and the SEM cross-section of the visible light region sample, please see Figure S1 in the Supplementary Materials.)

**Figure 2:**
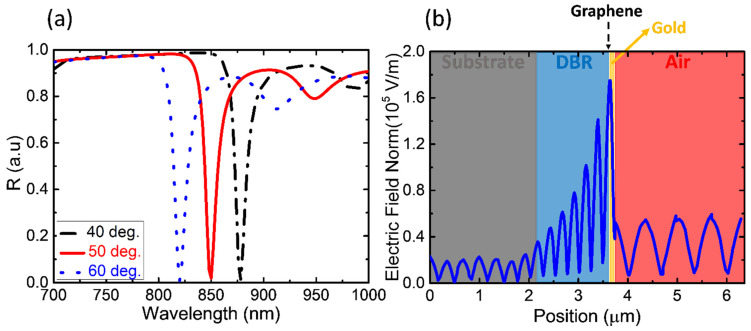
For Device A, (**a**) simulated TPP spectrum at the incident angles of 40°, 50°, and 60°, TM polarization. (**b**) Simulation of electric field distribution of the graphene–TPP structure at the incident angle of 50° of TM polarized 850 nm light source.

Tamm Plasmonic structures have been extensively investigated; we can finely tune the wavelength of the target resonance by changing the incident angle or the thickness of the top dielectric layer of the DBR, and we can also change the different metal films (e.g., Au, Ag, Al) to achieve a higher quality factor (*Q*-factor) of the TPP resonance. The TP structure and excited TPP resonance were designed using the finite-element method in COMSOL Multiphysics (5.3, Stockholm, Sweden). In this model, a plane-wave light source of 700–1000 nm obliquely illuminates the designed TP structure, and it is transverse magnetic (TM) polarization. The different simulated TPP resonance peaks are as shown in Figure 2a. A TPP resonance with a wavelength of 850 nm was created by a TM polarization incidence of 50°. The simulated electric field distributions are shown in Figure 2b, where a TM-polarized light at a fixed wavelength of 850 nm is obliquely incident on the TP structure. The strongest field is located at the metal–DBR interface where the graphene channel is established. (For the simulated field distributions of Device B, and the visible light region sample, please see Figure S2).

**Figure 3:**
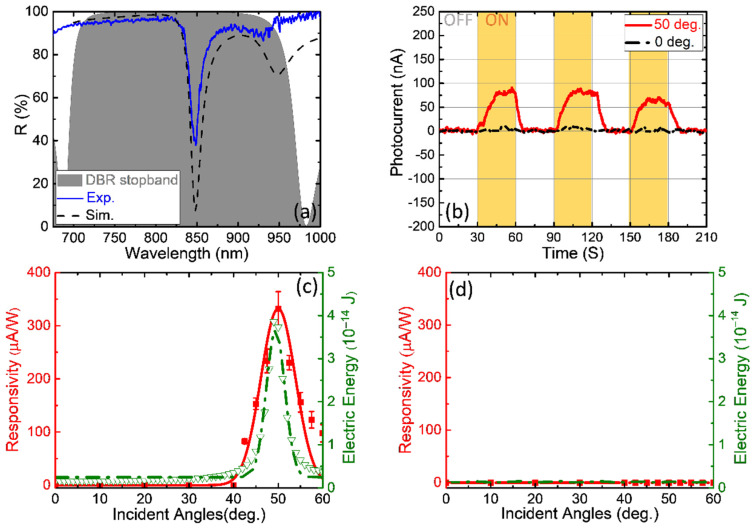
(**a**) TPP spectrum of Device A of the experiment and the simulation in the DBR stopband area (gray area) at the incident angle of 50°, TM polarization. (**b**) TM polarization photocurrent measurements of Device A; the solid red and black dash-dot lines indicate the incident angles of the 850 nm CW laser light source of 50° and 0°, respectively. (Yellow and white areas indicate the laser ON and OFF). (**c**) Different responsivities and simulated electric energy values at the incident angles of the light source from 0–60°, which is noted as the experimental group. (**d**) The control group results (W/O TPP sample) compared to (**c**).

Figure S3a shows the TPP resonance of Device A, the NIR-region sample, which is at a wavelength of 945 nm under a normal-incidence halogen light source. When the incident angle was increased, the resonance peak shifted to a shorter wavelength (blue shift). In Figure 3a, Device A is obliquely illuminated (50°) by a TM-polarized halogen light source on a rotational platform and detected by a spectrometer (Ocean Optics USB2000, Orlando, FL, USA), and the wavelength of the measured TPP resonance (blue solid line) can fit to that of the simulated TPP resonance (dash-dotted black line); however, the difference in reflectance between the experiment and simulation can be observed, which is caused by the error in the thickness of the metal film in the fabrication process. (The simulated reflection spectra of the structure considering the inhomogeneities of the thickness of Device B are presented in Figure S4b).

Figure 3b depicts the different photocurrent intensities of Device A measured by a source meter (Keysight B2901A, Santa Rosa, CA, USA), as a TM-polarized, 850 nm continuous wave (CW) laser beam was incident on the sample at 0° and 50°. At an incident angle of 50°, the TP structure can absorb most of light at this wavelength; this leads to an average photocurrent of ~80 nA, which is greater than that at normal incidence (0°). The green dash-dotted curve in Figure 3c illustrates the simulated electric energy of the TP structure at incident angles ranging from 0° to 60°, and the photocurrent responsivity (*R* = photocurrent intensity (*I***_PC_**)/Light source power (*P***_light_**)), the red solid curve in Figure 3c, has a specific peak corresponding to the electric-energy curve. The maximum value of the responsivity is ~330 μA⋅W^−1^ when the incident light power is 0.24 mW. Although both the peaks from the simulation and experiments happen at the same angle of 50, it can be noticed that there is still little deviation between the red and green curves because the simulated TPP (green curve) owns a better-quality factor in resonance than the measured TPP (red curve). Next, we removed the DBR layer to create a W/O TPP sample as a control group (for a detailed schematic of the control group, please see Figure S3b). The result is shown in Figure 3d, and it can be seen that there is no specific simulated electric-energy peak that affects the photocurrent responsivity if the TP structure does not exist. By comparison with the experimental and control groups (Figure 3c,d), one can see that the photocurrent responsivity is influenced by the absorption of the structure, and we introduce this graphene–TPP structure (Device A) to an angle-selective device.

### 3.2. Wavelength Selectivity (Device B)

**Figure 4:**
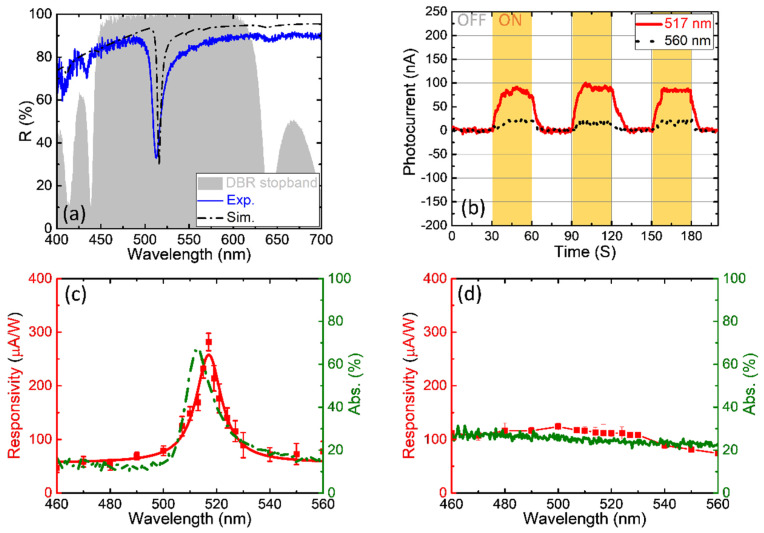
(**a**) TPP spectrum of Device B of the experiment and the simulation in the DBR stopband area (gray area) at normally incident. (**b**) Photocurrent measurements of Device B, the solid red and black dot lines indicate the wavelength of a light source of 517 nm and 560 nm, respectively. (Yellow and white areas mean the light source ON/OFF). (**c**) Different responsivities and reduced absorption percentages at the wavelength of the light source from 460–560 nm, which is noted as the experimental group. (**d**) The control group results (W/O TPP sample) compared to (**c**).

As we have already discussed the angle selectivity in Section 3.1, in this section, we introduce a wavelength-dependent device, Device B, which is a visible-light-region sample. As shown in Figure 4a, Device B was normally illuminated by a halogen light source passing through the objective lens (whose numerical aperture, N.A. = 0.13) of our microscope (Olympus BX51, Shinjuku, Tokyo, Japan), and the TPP resonance (blue solid curve) was measured using the same spectrometer as for Device A. In the DBR stopband area, the experimental TPP data fit the simulated data, which are shown as black dash-dotted curves. (The simulated reflection spectra of the structure considering the inhomogeneities of the thickness of Device A are presented in Figure S4a).

Figure 4b shows the different photocurrent intensities of Device B, as a light source coming from a monochromator that is illuminated by xenon light incident on the sample at wavelengths of 517 nm and 560 nm. A wavelength of ~517 nm is the most efficiently absorbed by the structure, which causes an average photocurrent of 87 nA, which is approximately 4.3 times greater than that at a wavelength of 560 nm when the absorption reduces from 65% (517 nm) to 15% (560 nm). In Figure 4c, the green dash-dotted curve shows the reduced absorption of the TP structure at wavelengths of the light source from 460 to 560 nm, and the photocurrent responsivity (solid red curve) has a specific peak in the absorption curve. The maximum value of the responsivity is approximately 271 μA⋅W^−1^ when the power of the 517 nm incident light is 0.32 mW. We also prepared a W/O TPP sample as a control group (for a detailed schematic of the control group, please see Figure S4). In Figure 4d, there is no specific absorption peak that affects the photocurrent responsivity; therefore, the spectrum is a smooth line. Comparing the experimental and control groups (Figure 4c,d), we consider Device B to be a wavelength-selective device.

## 4. Conclusions

The contribution of this work is the simplification of the structure of the photodetector device by stacking layers to form the plasmonic structure. The results confirm that our Graphene–TPP photodetector can be universally applied to various optoelectronic materials. With the field confinement located at the metal–DBR interface, the TPP signal can be detected and converted into electrical signals.

In conclusion, a Graphene–TPP photodetector device was developed. By illuminating the TP structure, a strong localized field was generated by the structure and detected by graphene, which converted to the photocurrent response. Different photocurrent responsivities were observed at different incident angles and light source wavelengths. The responsivity of the photodetector can be affected by the absorption of the structure. In Device A, the responsivity could be enhanced from approximately 0 mA/W to 330 μA⋅W^−1^ at an incident angle of 50°, as the absorption increased from 0% to 60%; hence, it can be noted as an angle-selective device. On the other hand, the responsivity in Device B was enhanced from 60 mA/W to 271 μA⋅W^−1^ at the wavelength of the light source of 517 nm, as the absorption increased from 15% to 65%; therefore, it can be considered as a wavelength-selective device. Additionally, two devices working in the visible and NIR regions were demonstrated, indicating that the graphene–TPP device can work in both regions.

To enhance responsivity, the graphene layer in the developed device can be replaced by materials such as WS_2_ and MoS_2_, and the wavelength and angle selectivity can be applied to next-generation miniature LiDAR detecting, sun sensors, and laser beacon tracking and navigational instruments.

## Data Availability

The data presented in this study are available on request from the corresponding author.

## Supplementary Materials

The following supporting information can be downloaded at: https://www.mdpi.com/article/10.3390/nano13040693/s1, Figure S1: (a) and (b) indicate the schematic of Device B and the SEM cross-section of the DBR. The fabricating methods are the same as Device A with a deposited 50-nm TPP silver film. Among DBR dielectric layers, the designed thickness of SiO_2_ (TiO_2_) is ≈ 83.7 nm (≈ 49.3 nm), thus the central wavelength of DBR *λ*_c_ = 500 nm, Figure S2: For Device A, (a) simulated TPP spectra and (b) simulation of electric field distribution at a fixed wavelength of 517 nm light source. The inset below depicts a 2D-mapping of electric field distribution in our simulated TPP structure, Figure S3: For Device A, (a) the reflection spectra of the TPP signal under normal incidence light source and (b) the schematic of the control group (W/O TPP sample), the blue layer is TiO_2_ noted as the toppest dielectric layer of the DBR contacting with graphene, Figure S4: Measured and simulated reflectance spectra of the Device A (a) and Device B (b).
