# Supplementary material for: Wavelength- and Angle-Selective Photodetectors Enabled by Graphene Hot Electrons with Tamm Plasmon Polaritons
Source: Nanomaterials (Basel). 2023 Feb 10;13(4):693. doi: 10.3390/nano13040693 (PMC9961655; doi:10.3390/nano13040693)
Supplement: Supplementary file 1 [file nanomaterials-13-00693-s001.zip › nanomaterials-2172391-supplementary.pdf]

nanomaterials

Supplementary Materials

Wavelength- and Angle-Selective Photodetectors Enabled by Graphene Hot Electrons with Tamm Plasmon Polaritons

Cheng-Han Huang [1], Chia-Hung Wu [2], Rashid G. Bikbaev [3,4], Ming-Jyun Ye [2], Chi-Wen Chen [1], Tung-Jung Wang [5], Ivan V. Timofeev [3,4], Wei Lee [5] and Kuo-Ping Chen [5,6,*]

1 Institute of Photonic System, National Yang Ming Chiao Tung University, 301 Sec. 2, Gaofa 3rd Road, Tainan 711010, Taiwan
2 College of Photonics, National Yang Ming Chiao Tung University, 301 Sec. 2, Gaofa 3rd Road, Tainan 711010, Taiwan
3 Kirensky Institute of Physics, Federal Research Center KSC SB RAS, 660036 Krasnoyarsk, Russia
4 Siberian Federal University, 660041 Krasnoyarsk, Russia
5 Institute of Imaging and Biomedical Photonics, National Yang Ming Chiao Tung University, 301 Sec. 2, Gaofa 3rd Road, Tainan 711010, Taiwan
6 Institute of Photonics Technologies, National Tsing Hua University, Hsinchu 30013, Taiwan
* Correspondence: kpchen@ee.nthu.edu.tw

Visible Light Region Sample (Device B)

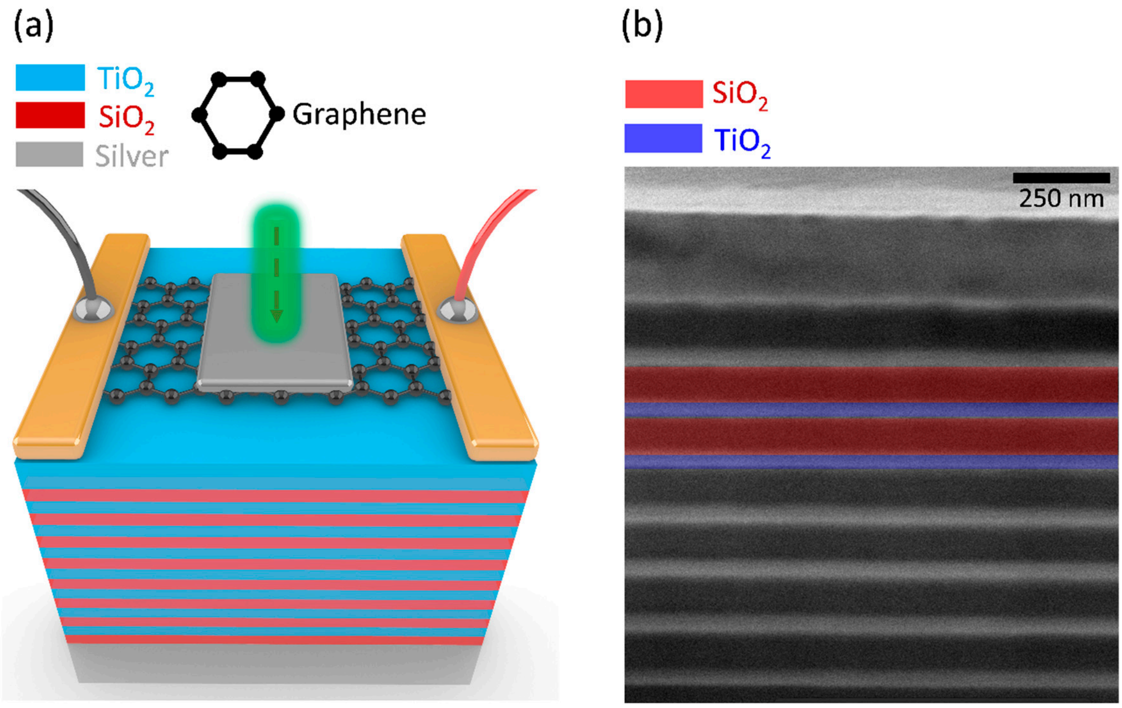

Figure S1. (**a**) and (**b**) indicate the schematic of Device B and the SEM cross-section of the DBR. The fabricating methods are the same as Device A with a deposited 50-nm TPP silver film. Among DBR dielectric layers, the designed thickness of SiO_2 (TiO_2) is ≈ 83.7 nm (≈ 49.3 nm), thus the central wavelength of DBR λ_c = 500 nm.

In the COMSOL simulation results below, a plane wave of 400–700 nm light source was normally illuminated on the designed TP structure. Figure S2 (a) shows the reflection spectra of the TPP signal, the resonance wavelength is at 517 nm. The simulated electric

fields distributions are shown in Figure S2 (b), a fixed wavelength of 517 nm light source was incident on the TP structure. The strongest field also locates at the metal-DBR interface where the graphene channel is fabricated.

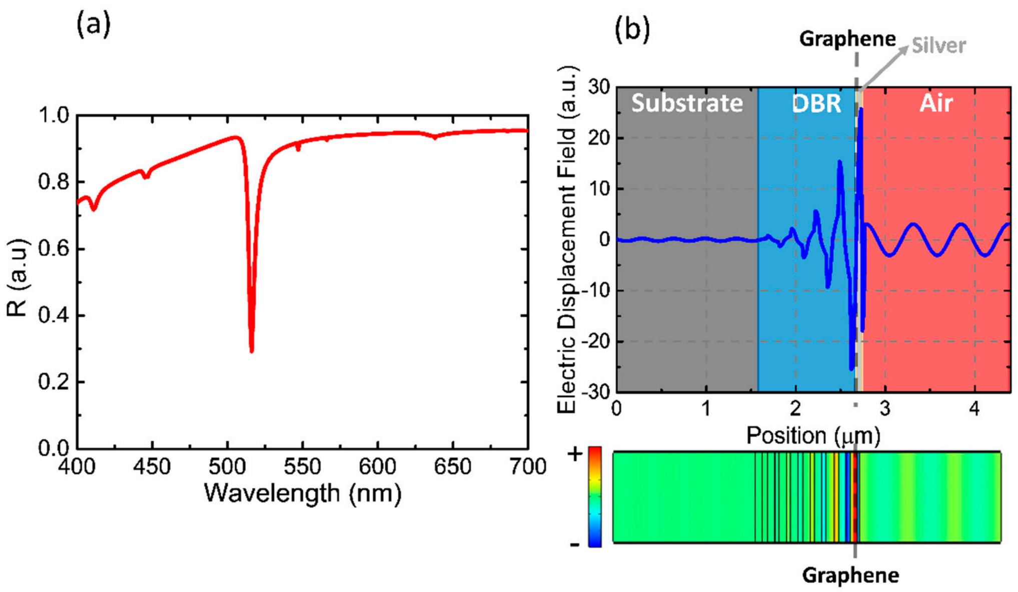

Figure S2. For Device A, (**a**) simulated TPP spectra and (**b**) simulation of electric field distribution at a fixed wavelength of 517 nm light source. The inset below depicts a 2D-mapping of electric field distribution in our simulated TPP structure.

Measurement

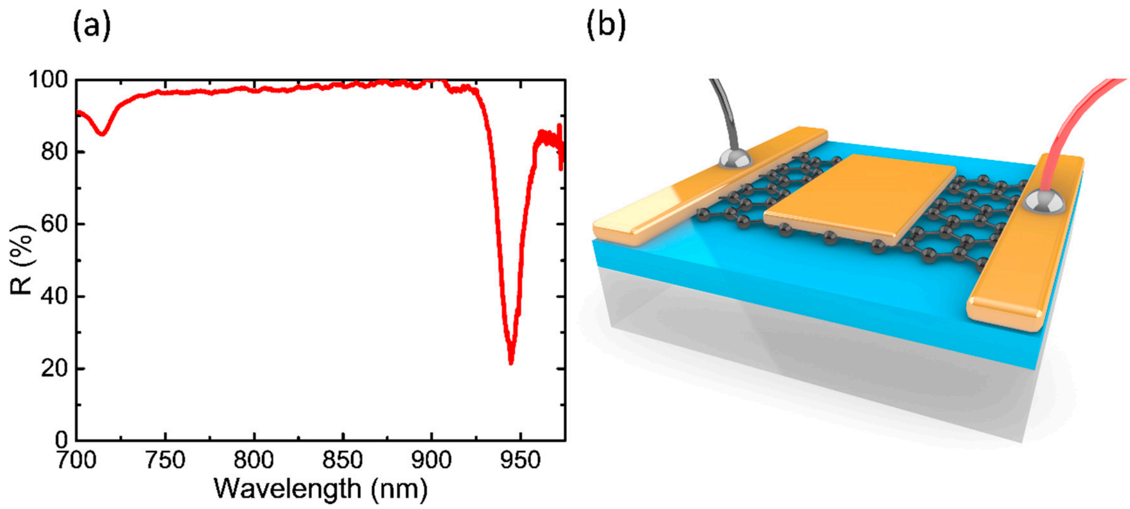

Figure S3. For Device A, (**a**) the reflection spectra of the TPP signal under normal incidence light source and (**b**) the schematic of the control group (W/O TPP sample), the blue layer is TiO_2 noted as the toppest dielectric layer of the DBR contacting with graphene.

Transfer-matrix Simulation

The reflectance, transmittance and absorptance spectra of the structure can be calculated using the transfer-matrix method. The transfer matrix $\widehat{M}$ of the entire structure that relates the amplitudes of the incident and transmitted waves is a product of 2 × 2 matrices:

$$\widehat{M} = \widehat{T}_{01}\widehat{T}_{02}\cdots\widehat{T}_{N-1,N}\widehat{T}_{N,S} \quad (1)$$

Where the transfer matrix of each layer is

$$\hat{T}_{n-1,n} = \frac{1}{2}\begin{pmatrix}(1+h)e^{-i\alpha_n\gamma_n} & (1-h)e^{i\alpha_n\gamma_n}\\(1-h)e^{-i\alpha_n\gamma_n} & (1+h)e^{i\alpha_n\gamma_n}\end{pmatrix} \quad (2)$$

Here, $h=(\varepsilon_n/\varepsilon_{n-1})^{1/2}$, ε_n is the permittivity of the n-th layer, $\alpha=(\omega/c)\varepsilon_n^{1/2}$, ω is the wave frequency, c is the speed of light, and $\gamma_n = z_n - z_{n-1}$ is the layer thickness, where $n = 1,2,\ldots, N$ and z_n is the coordinate of the interface between the n-th and $(n + 1)$ layers. The transfer matrix for the orthogonally polarized wave is obtained by substituting $(\varepsilon_{n-1}/\varepsilon_n)^{1/2}$ for h. The transmittance (T), reflectance (R) and absorbance (A) are determined as:

$$T(\omega)=\frac{1}{\left|\hat{M}_{11}\right|^2},\quad R(\omega)=\frac{\left|\hat{M}_{11}\right|^2}{\left|\hat{M}_{21}\right|^2},\quad A(\omega)=1-T(\omega)-R(\omega). \quad (3)$$

Here, $\hat{M}_{11}$ and $\hat{M}_{21}$ are the elements of matrix $\hat{M}$.

The transfer-matrix method provides exact solution for simulation of one-dimensional (layered) structures and allows to perform a large number of calculations in a short period of time. In this regard, we used this method to vary the parameters of the structure, ensuring the better agreement of the simulation result with experimental data. Often, the broadening of the spectral line is induced by a violation of the plane-parallelism of the photonic crystal layers, the beam diameter, or some deviation from the normal when measuring the reflection spectra of the structure through the objective. Taking into account these factors with averaging over a large number of calculations makes it possible to achieve a good agreement of calculations with experimental data. As a demonstration, we calculated the reflection spectra of a photonic crystal conjugated with metal films, taking into account the inhomogeneities of the thickness of the first layer of the photonic crystal.

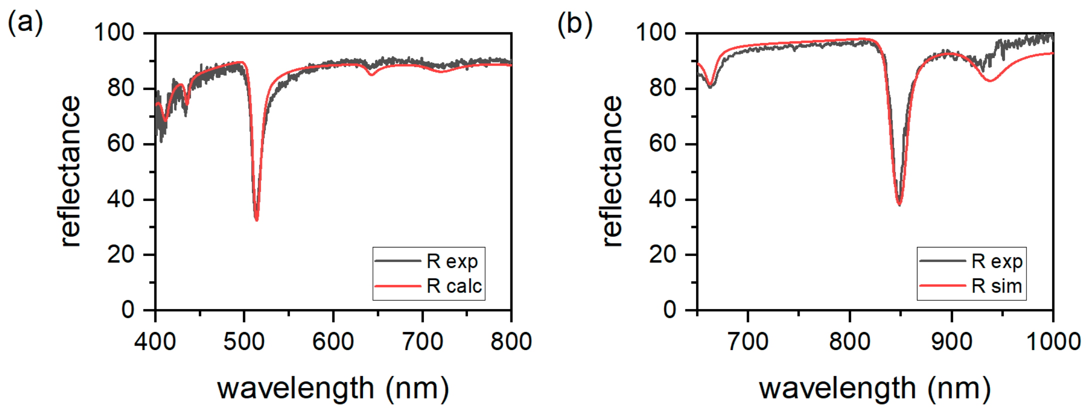

Figure S4. Measured and simulated reflectance spectra of the Device A (**a**) and Device B (**b**).

The calculation results are shown in Figure S4. Taking into account the inhomogeneities in the thickness of the first layer of the photonic crystal makes it possible to achieve good agreement with the measured spectra. In this case, the inhomogeneities were taken into account as a 10% thickness deviation of the first layer of photonic crystal, and the averaging of spectra was carried out according to the normal distribution.

Calculation of Quantum Efficiency

According to the equation of the external quantum efficiency (EQE): $R \times \frac{1240}{\lambda}$; R is responsivity and λ is the operating wavelength. Based on the calculation, our device A (NIR, angle-dependent device) owns EQE of 0.05%, and our device B (visible light, wavelength-dependent device) owns EQE of 0.065%.
